# Alterations in the Gut Microbiota of Tibetan Patients With Echinococcosis

**DOI:** 10.3389/fmicb.2022.860909

**Published:** 2022-05-09

**Authors:** Deping Cao, Mingquan Pang, Defang Wu, Gen Chen, Xiaohong Peng, Kai Xu, Haining Fan

**Affiliations:** ^1^The Department of Human Parasitology, Basic Medical College of Guilin Medical University, Guilin, China; ^2^Department of Hepatobiliary and Pancreatic Surgery, Affiliated Hospital of Qinghai University, Xining, China; ^3^The Key Echinococcosis Laboratory, Affiliated Hospital of Qinghai University, Xining, China

**Keywords:** Qinghai-Tibetan Plateau, cystic echinococcosis, alveolar echinococcosis, gut microbiome, alteration

## Abstract

There are two main types of echinococcosis, namely alveolar echinococcosis (AE) and cystic echinococcosis (CE). They are zoonotic parasitic diseases caused by the metacestodes of *Echinococcus multilocularis* and *Echinococcus granulosus*. In order to explore the gut microbiome composition of patients with echinococcosis, we analyzed fecal samples of seven patients with AE, six patients with CE, and 13 healthy individuals from the Qinghai-Tibetan Plateau, China. Using metagenomic next-generation sequencing, we identified fecal bacteria in the patients with AE and CE. The gut microbiota was analyzed by next-generation metagenomic sequencing (mNGS) to compare patients with either AE or CE against healthy individuals. We found there were some differences between them in abundant bacteria. Our results led to five findings: (1) Between patients with echinococcosis and healthy individuals, the differential bacteria were from four phyla: Firmicutes, Proteobacteria, Bacteroidetes, Actinobacteria. (2) *Rothia mucilaginosa*, *Veillonella dispar*, *Veillonella atypica*, *Streptococcus parasanguinis, Streptococcus salivarius*, and *Alistipes finegoldii* were abundant in the feces of patients with AE. (3) *Bacteroides dorei, Parabacteroides distasonis, Escherichia* sp_E4742, and *Methanobrevibacter smithii* were abundant in the feces of the patients with CE. (4) At the phylum and class level, compared to the AE group, the healthy group was characterized by higher numbers of Actinobacteria. (5) At the family level, Lachnospiraceae and Eubacteriaceae were more abundant in the feces of healthy individuals than in AE patients. The genera *Coprococcus*, *Eubacterium*, and *Bilophia* were more abundant in the healthy group, while the genus *Rothia* was more abundant in the AE group. The results of this study enrich our understanding of the gut microbiome composition of patients with AE and CE in the Qinghai-Tibetan Plateau.

## Introduction

Echinococcoses are zoonotic parasitic diseases caused by the metacestodes of *Echinococcus multilocularis* and *Echinococcus granulosus*; they infect humans, herbivorous livestock, and rodents. There are two primary types of echinococcosis: alveolar echinococcosis (AE) and cystic echinococcosis (CE). Both types are now considered global public health and socio-economic problems (McManus et al., 2012; Wen et al., 2019). Echinococcosis is most frequently found in the vast pastoral and semi-agricultural areas of northwest and southwest China such as the Qinghai-Tibetan Plateau where a high incidence of mixed echinococcosis infections has been reported (Wang et al., 2014). The route of infection is mainly the ingestion of eggs or gravid proglottids of *E. multilocularis* and *E. granulosus* that have been excreted in canid feces (from foxes, wolves, and dogs). AE is considered a risk factor for the development of malignant tumors—a disease known as “bug cancer,” which can invade other organs such as the lung and brain through direct spread and blood metastasis. The prognosis of this type of echinococcosis is extremely poor, with a 10-year fatality rate of up to 90% (Ren et al., 2007; Kern et al., 2017).

The gut microbiome can be regarded as an essential “organ” in the human body, and intestinal bacteria have been a popular research focus in recent years in the fields of microbiology, medicine, and genetics. As the largest microbial reservoir in the human body, the gut microbiome interacts with the mucosal immune system and plays an important immunomodulatory role (Chinen and Rudensky, 2012). The liver is the body’s largest digestive organ, and approximately 70% of the liver blood supply comes from the hepatic portal vein. This particular anatomical feature (the liver–intestinal axis) causes the liver to be exposed to a large number of bacteria and their products (Gui et al., 2021). Therefore, a key link exists between the intestinal microecosystem and the pathogenesis and prognosis of many diseases (Dai and Li, 2021). Yooseph et al. (2015) found that composition of the gut microbiota can affect the risk of *Plasmodium falciparum* infection in malaria endemic areas. Schachter et al. (2020) reported that parasitism of *Trichuris trichiura*, an intestinal soil-derived helminth, can alter the intestinal microbiota composition and induce bacterial invasion of the large intestine epithelium. Many studies have shown that increased intestinal permeability and changes in intestinal flora diversity and structure are characteristics of many chronic liver diseases and promote their development (Bajaj et al., 2014; Bhat et al., 2016). A clear correlation has been found between the gut microbiota and *S. japonicum and S. mansoni* infection in laboratory mice and humans (Schneeberger et al., 2018; Zhao et al., 2019). The translocation of bacteria and bacterial pathogen-associated molecular patterns (PAMPs) is common in chronic liver diseases and in fibrosis during chronic liver injury (Seki et al., 2007; Dapito et al., 2012). When the liver is continually exposed to metabolites of intestinal microbiota, TLRs are activated in target cells by lipopolysaccharides and induce inflammatory signaling pathways that lead to repeated damage to liver cells, subsequently causing liver fibrosis, cirrhosis, and even hepatocellular carcinoma (Yooseph et al., 2015; Zhou et al., 2017). Anter et al. (2020) found that there is a correlation between curcumin dosages and the species of gut microbiota in mice infected with *S. mansoni*.

The first immune defense layer comprises mechanical, chemical, and microbiological barriers when microorganisms enter the host body. Mucus includes antimicrobial peptides secreted by the gut epithelium in the gastrointestinal tract to bind and entangle pathogens (Martin et al., 2010; Wang L. et al., 2017). Numerous studies have confirmed that the intestinal microbiota transmit signals to distal organs through metabolites, linking bacteria to the immune system, endocrine system, nervous system, host metabolism, and other physiological functions (Unger et al., 2016; Zhang et al., 2021). An abundance of gut microbes was found in patients with liver cirrhosis, with 54% originating from the mouth. Oral *Streptococcus salivarius* and *Veillonellaceae* species are abundant taxa that prompt the emigration of bacteria from the mouth to the intestine to play an important role in the process of liver cirrhosis. *S. salivarius* and *Veillonellaceae* can thus be used as microbial markers for the diagnosis of liver cirrhosis (Qin et al., 2014).

Almost 100% of AE and approximately 70% of CE cases occur in the liver, with these parasites invading through the intestinal wall. This suggests that changes in the intestinal flora are correlated with the occurrence of echinococcosis. In fact, Bao et al. (2018) found that changes in the intestinal bacteria of mice with CE might be related to metabolic pathways. We conducted this study to determine the characteristics of the intestinal flora in patients with AE and CE.

## Subjects and Methods

### Subjects

Thirteen stool samples from 13 Tibetan patients with CE or AE were collected at Qinghai University Hospital between October 2020 and December 2020; six fecal samples were from patients with CE and seven were from patients with AE. Thirteen fecal samples from accompanying family members were used as controls. The study was approved by the Ethics Committee of Qinghai University Hospital (approval number: P-SL-2019054), and all subjects provided informed consent to participate and signed an informed consent form.

#### Inclusion and Exclusion Criteria

The inclusion criterion for the study were as follows: (1) The diagnostic criteria of AE and CE were based on “Expert consensus on diagnosis and treatment of hepatic cystic and alveolar echinococcosis” (2019 edition) (Echinococcosis Surgical Committee Surgeons Branch Chinese Medical Association, 2019). (2) Diagnosis and staging of patients with AE and CE depended on imaging—primarily ultrasonic (US), magnetic resonance (MRI), and computed tomography (CT) imaging. To a large extent, serology played a confirmatory role. (3) Individuals lived in the pastoral area of Qinghai Tibetan plateau. The exclusion criteria for the study were as follows: (1) history of abdominal surgery; (2) intestinal dysfunction; (3) food allergies; (4) the use of antibiotics or probiotics during the past 3 months. With consideration to the inclusion criteria, for the control group we chose the patient’s family members to ensure comparable diet and other lifestyle habits. For sample collection, fresh stool samples of participants in the AE and CE groups and those from the healthy individuals were collected in high-pressure aseptic feces collectors and stored at −80°C.

### Metagenome Sequencing

#### DNA Isolation From Feces

DNA was extracted from 180 to 200 mg of solid feces as previously reported (Costea et al., 2017) using a Nucleic acid Extraction and Purification Kit (NO: DR-HS-A010, Guangzhou Da’an Gene Co., Ltd, Guangzhou). DNA was quantified with the Qubit Fluorometer 2.0 (ThermoFisher Scientific, Waltham, MA, United States).

#### cDNA Library Construction

The RNA in the total DNA was treated with reverse transcriptase and DNA polymerase to synthesize double-stranded cDNA. The cDNA fragments were broken to 150–300 bp *via* a fragmentation enzyme, and the ends were connected to adaptors with DNA ligase to construct a library for sequencing. cDNA library construction was performed following the manufacturer’s protocol. All kits needed for library construction (BY-WK-001) were supplied by Guangzhou Da’an Gene Co., Ltd. The library quality was assessed using a Qubit Fluorometer 2.0 and an Agilent Bioanalyzer 2100 system (Agilent in Life Sciences, Santa Clara, CA, United States).

#### Computer Sequencing

The sequencing library was amplified by emulsion PCR to form the sequencing template, and the positive template was enriched to standard sequencing requirements. Micropores DNA was loaded into of semiconductor chips and, using single-stranded DNAs as a template, complementary DNA strands were synthesized with DNA polymerase according to the principle of base complementarity. For each base-pair extension of the DNA, one proton was released, resulting in local pH changes. Ion sensors detected pH changes and converted chemical signals into digital signals, allowing the bases to be read in real time, and finally to obtain the base sequence of each DNA fragment (DR-CX-A001, Universal Semiconductor Sequencing Kit, Guangzhou Da’an Gene Co., LTD, Guangzhou). The Ion Torrent high-throughput sequencing DA8600 platform (Guangzhou Da’an Gene Co., LTD) was used for sequencing, and the raw data obtained from the sequencing was used for post-information analysis.

### Data Analysis

#### Metagenomic Sequencing Data Analysis

Raw data were analyzed by Da’an Bio-technology Co. Ltd. (Guangzhou, China). Raw data was sorted and filtered by splitting samples according to sequencing labels, removing adaptors, and filtering low-quality sequences. Host sequence removal, pathogen database alignment, and annotation were based on the comparison database. Sequences were clustered into operational taxonomic units (OTUs) with a cutoff value of 97% using UPARSE software. Each OTU corresponded to a representative sequence. BWA software^1^ was used to analyze the sequencing data. Kaiju software (Menzel et al., 2016) was used to classify clean reads into species as a complement to the non-redundant database-based species annotation method.

#### Bacterial Diversity Analysis

The total abundance and alpha and beta diversity of microbial communities were estimated by MOTHUR software.^2^ Qiime software was used to calculate Chao1, Shannon, and Simpson indices. The Chao1 index represents the total number of species in the sample community without considering the abundance of each species in the community; the Simpson index reflects the probability of two randomly selected individuals belonging to different species; the Shannon index reflects the species diversity of sample communities, and is affected by species richness and evenness. β-Diversity (between-sample diversity) focuses on OTU diversity comparison of different samples. PCoA ANOVA was used to analyze the differences of the OTU data between AE, CE, and healthy individuals on the two-dimensional coordinate map to observe the taxonomic difference of microbial community diversity among different groups and the bacterial species closely related to disease.

#### LefSe Analysis

Taxonomic characterization was performed using a LDA (linear discriminant analysis) with a calculation of the LefSe (LAD effect size). The figure shows species with significant differences in abundance between different groups when the LDA score is greater than the set value (the default setting is 2).

#### Carbohydrate Active Enzyme Annotation

dbCAN2 meta server online software was used to analyze Carbohydrate Active Enzymes (CAZymes) in the gut microbiome of AE, CE, and healthy individuals (dbCAN)^3^ (Yin et al., 2012).

#### Statistical Analysis

The non-parametric Kruskal–Wallis rank-sum test, Wilcoxon rank-sum test and chi-square test were used. The threshold for the LDA score was >2, representing the criterion for identifying significant biomarkers.

## Results

### General Condition of the Patients

A summary of patient characteristics is provided in Table 1. There are seven patients with AE, six patients with CE and 13 healthy individuals.

**TABLE 1:** General condition and clinical liver function index of 13 patients with echinococcosis.

Patient No.	M/F	Age	AE/CE	ALT (U/L)	ALP (U/L)	AST (U/L)	TP (g/L)	ALB (g/L)	Te (umol/L)	UIBC (umol/L)
Patient 1	F	31	AE	22	193	23	72.8	30.6	5.1	30.2
Patient 2	M	43	CE	16	88	19	76.8	43.6	15.53	32.6
Patient 3	F	44	AE	55	405	32	77.2	36.5	12.1	17.4
Patient 4	F	53	CE	15	73	19	73.2	36.2	9.09	36.5
Patient 5	F	31	AE	25	105	31	78.9	38.0	5.2	65.2
Patient 6	F	24	CE	15	90	17	60.9	35.6	3.0	53.5
Patient 7	F	38	AE	38	49L	25	63.9	36.3	18.77	45.6
Patient 8	F	28	AE	28	130	23	59.3	33.8	4.18	51.8
Patient 9	M	15	AE	57	144	69	65.5	29.4	6.63	14.1
Patient 10	F	25	AE	21	72	16	71.6	38.3	11.4	32.5
Patient 11	M	35	CE	28	551	54	84.3	26.0	8.6	12.9
Patient 12	M	48	CE	27	165	23	69.8	41.3	10.83	40.2
Patient 13	M	51	CE	66	299	28	68.3	35.6	9.51	37.5

### Comparison of the Gut Flora in Samples

**FIGURE 1:**
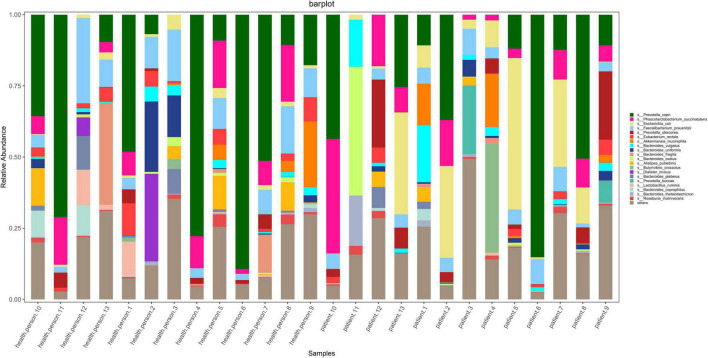
Stacked bar diagram of species abundance for each sample at the level of the species including seven patients with alveolar echinococcosis (AE), six patients with cystic echinococcosis (CE), and 13 healthy individuals. They are: *Prevotella copri, Phascolarctobacterium succinatutens, Escherichis coli, Faecalibacterium prausnitzli, Prevotella stercorea, Eubacterium rectale, Akkermansia muciniphila, Bacteroides vulgatus, Bacteroides uniformis, Bacteroides fragilis, Bacteroides ovatus, Alistipes putredinis, Butyrivibrio crossotus, Dialister invisus, Bacteroides plebeius, Prevotella buccae, Lactobacillus ruminis, Bacteroides coprophilus, Bacteroides thetaiotaomicron, Roseburia inulinivorans.*

**FIGURE 2:**
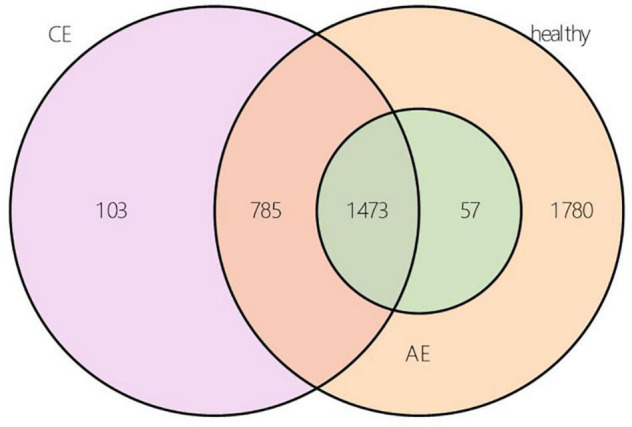
Operational taxonomic unit (OUT) Venn diagram of alveolar echinococcosis (AE) group, cystic echinococcosis (CE) group, and healthy group in species level.

According to the results of OTU annotation, a histogram of the relative abundance of the top 20 species in each sample at the genus classification level was plotted. Although the top 20 bacteria in each sample were roughly same, the abundance of bacteria was different among samples (Figure 1). Venn diagram can intuitively represent the number of shared and unique OTUs in three samples. In this study, a total of 4198 OTUs numbers were found in the AE, CE, and healthy groups. Fifty seven, 103, 1780 OTUs numbers were unique to the AE group, CE group and the control group, respectively. indicating differences in species distribution between these three groups (Figure 2).

### Analysis of Species Differences Between Groups

#### LefSe Analysis

**FIGURE 3:**
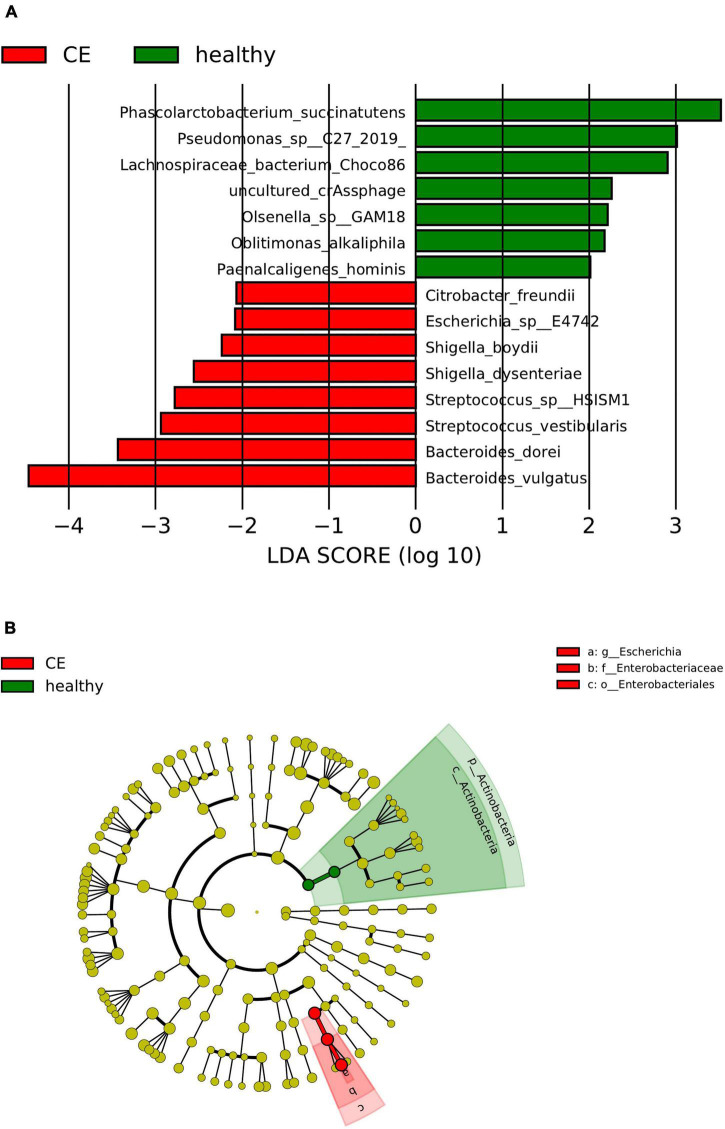
Panel **(A)** is the histogram of distribution with linear discriminant analysis (LDA) value ≥ 2. Panel **(B)** shows the evolutionary cladogram. Histogram of linear discriminant analysis (LDA) effect size (LEfSe) value distribution and evolutionary cladogram between patients with cystic echinococcosis (CE) and healthy individuals. **(B)**. The cladogram the red and green nodes represented specific microorganisms relevant to healthy and CE patients of a cladogram. Each node represents a biomarker. The yellow nodes represent the microorganisms that did not play an important role in the different groups. The diameter of each node is proportional to the taxon’s abundance. The circles that radiate from the inside to the outside represent the classification level from phylum to species (o, order; f, family; g, genus; s, species).

**FIGURE 4:**
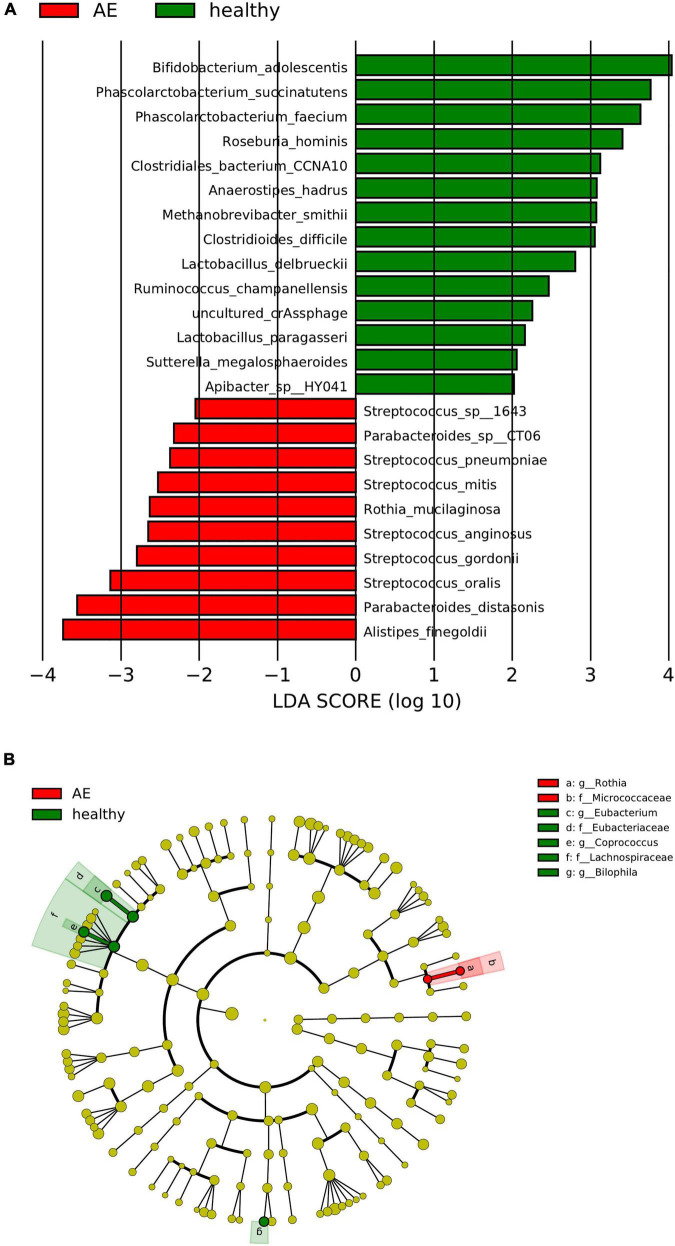
Panel **(A)** is the histogram of distribution with linear discriminant analysis (LDA) value ≥ 2. Panel **(B)** shows the evolutionary cladogram. Histogram of LDA effect size (LEfSe) value distribution and evolutionary clade between patients with CE and healthy individuals. g, genus; f, family; s, species; o, order. The meaning of the picture is the same as the one above.

The linear discriminant analysis (LDA) distribution diagram (LAD score > 2) showed an obvious alteration of the microbiota characterized by higher quantities of *Citrobacter freundii, Escherichia* sp_E4742, *Shigella boydii*, *Shigella dysenteriae*, *Streptococcus* sp_HSISM1, *Streptococcus vestibularis*, *B. dorei*, and *Bacteroides vulgatus* in the CE group. The LAD score of *B. vulgatus* was greater than four, indicating it is the most abundant bacteria in the CE group. Some bacterial species were abundant in the healthy group, including *P. succinatutens*, *Pseudomonas* sp_C27_2019, *Lachnospiraceae bacterium_Choco86*, *Olsenella* sp_GAM18, *Oblitimonas alkaliphila*, and *Paenalcaligenes hominis* (Figure 3A). The LAD cladogram revealed that in the healthy group the abundant bacteria were mainly distributed in the Actinobacteria phylum and Actinobacteria class, and in the CE group the abundant bacteria were mainly distributed in the Proteobacteria phylum and Enterobacteriales class (Figure 3B). There were some different abundant species in the AE group compared to the healthy group according to LefSe analysis. In the feces of patients with AE, the more abundant bacteria were *Rothia mucilaginosa*, *Veillonella dispar*, *Veillonella atypica*, *Streptococcus parasanguinis*, *S. salivarius*, and *Alistipes finegoldii*. *Lachnospiraceae* genera, *Eubacterium*, *Coprococcus*, and *Bilophila* at genus level and *Roseburia hominis*, *Bilophila wadsworthia*, *Eubacterium hallii*, and *Bifidobacterium adolescentis* were more abundant in the feces of healthy individuals than in that of patients with AE (Figures 4A,B). Moreover, *Streptococcus salivarius* and *Streptococcus salivarius*_unclassified might be more abundant bacteria in the AE group. In summary, the differential abundant bacteria belonged to the following four phyla: Firmicutes, Proteobacteria, Bacteroidetes, and Actinobacteria.

### α-Diversity and β-Diversity Analysis

**FIGURE 5:**
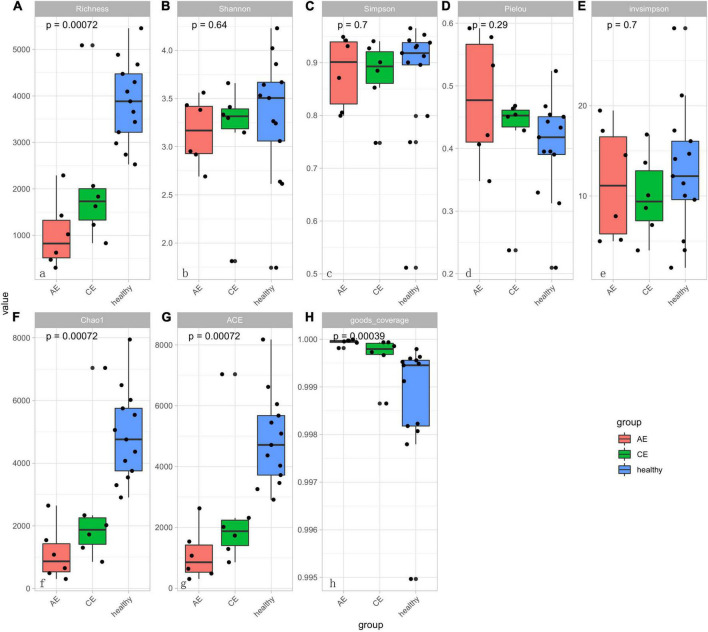
The diversity boxplot shows α-diversity in patients with alveolar echinococcosis (AE), patients with cystic echinococcosis (CE), and healthy people. Alpha-diversity estimations are similar for control, AE and CE groups. Results are presented as box and whiskers’ plots per group (AE, CE, and control) and per metric: **(A)** Richness; **(B)** Shannon index; **(C)** Simpson index; **(D)** Pielou; **(E)** Inv Simpson; **(F)** Chao1 index; **(G)** ACE index; **(H)** Good-coverage index. There were significant different in panels **(A,G,F,H)** (*P* < 0.001). That means the sample is rich in species. There was no significant differences of sample diversity between AE, CE, and healthy groups.

**FIGURE 6:**
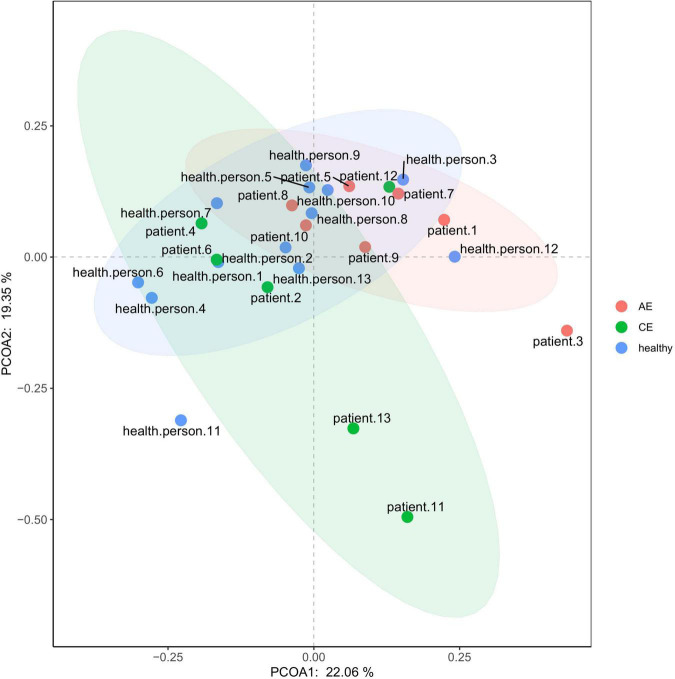
PcoA (principal co-ordinates analysis) analysis diagram.

There were differences in Chao1 and ACE (*P* < 0.00072, *P* < 0.00072) indexes of community richness (*P* < 0.00072) in terms of α-diversity among the gut microbiomes of patients in the AE, CE, and healthy groups. In terms of the Simpson, Shannon, and Invsimpson (*P* = 0.7, *P* = 0.64, *P* = 0.7, respectively) indexes of the diversity of the gut microbiome, there was no change in the diversity of gut microbiota, but there was a difference in the abundance of gut microbiota among these three groups (Figure 5). This was applied based on the relative abundances of sequences within communities and the extent of genetic divergence between sequences. Comparing microbiota variability using PCoA, the sample clustering effect of AE patients and CE patients was better, as there were differences among samples and good similarity within samples (Figure 6).

### Types of Carbohydrate Metabolism Genes per Group

**FIGURE 7:**
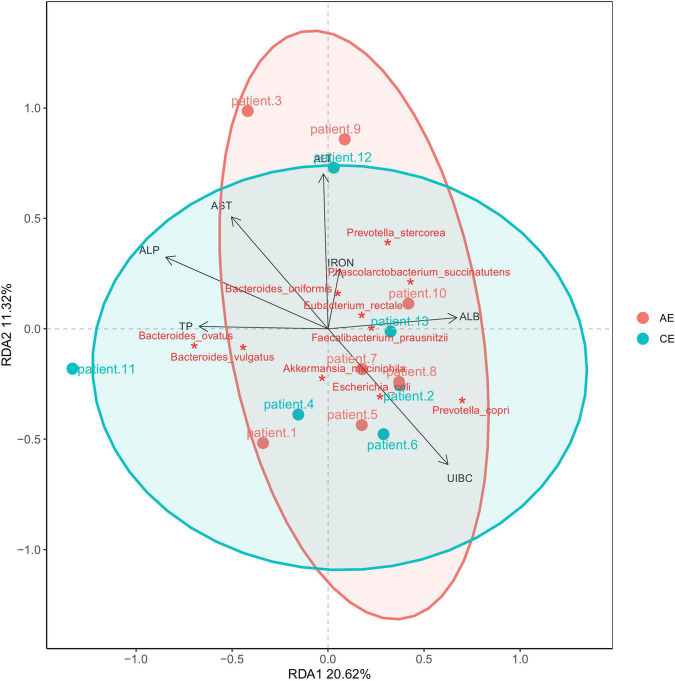
RDA analysis of blood alanine aminotransferase (ALT), aspartate aminotransferase (AST), IRON, serum casein (ALB), total protein (TP), unsaturated iron binding capacity (UIBC). It showed that UIBC was positive correlated with alveolar echinococcosis (AE) and cystic echinococcosis (CE) groups (R2 = 0.5592, Pr = 0.015, *P* < 0.05). There were no correlations for other clinical indexes—AST, IRON, ALT, TP, and ALB—between patients with AE and CE. Their Pr values were all greater than 0.05 (*P* > 0.05).

The carbohydrate enzymes of the gut microbiome were analyzed using CAZymes. Carbohydrate enzymes were divided into six categories: glycoside hydrolyses (GHs), glycoside transferases (GTs), carbohydrate esterases (CEs), polysaccharide lysozymes (PLs), carbohydrate binding modules (CBM), and coenzymes (AA). The GHs in patients with AE and the GTs in patients with CE were not different from those of healthy individuals. The Redundancy Analysis (RDA) diagram (Figure 7) showed that UIBC (unsaturated iron binding capacity) was positive correlated with AE and CE groups (R2 = 0.5592, Pr = 0.015, *P* < 0.05). There were no correlations for other clinical indexes—aspartate aminotransferase (AST), IRON, alanine aminotransferase (ALT), total protein (TP), and serum casein (ALB)—between patients with AE and CE. Their Pr values were all greater than 0.05 (*P* > 0.05). AST is related to *Ruminococcus torques*; IRON is related to *Phascolarctobacterium succinatutens*; ALT is related to *Sutterella wadsworthensis*, *Veillonella parvula*, and *V. atypica*; and ALB, TP, and UIBC are related to *Coprococcus eutactus*.

## Discussion

The healthy human intestinal microbiota consists mainly of members of the phyla Firmicutes and Bacteroidetes with relatively few members of the phyla Proteobacteria, Actinobacteria, Fusobacteria, and Verrucomicrobia (Eckburg et al., 2005). An increasing number of studies confirms that the quality of the gut microbiota plays an important role in the development of chronic liver disease (Qin et al., 2010; Minemura and Shimizu, 2015; Xu et al., 2020). In fact, the occurrences of many diseases, such as diabetes mellitus, metabolic syndrome, non-alcoholic fatty liver disease, chronic obstructive pulmonary disease, and cardiovascular diseases, are known to be related to disorders of the gut microbiome (Vijay-Kumar et al., 2010; Qin et al., 2012; Burcelin et al., 2015; Wang X. K. et al., 2017; Wu et al., 2021).

Most of the bacteria related to the liver function index belonged to Firmicutes according to the network diagram. Meanwhile, it was found that UCBI was related to *C. eutactus*, and that this correlation may be significant. Changes in the gut microbiota cause changes in intestinal iron metabolism, and abnormal iron metabolism will cause abnormal physiological functions of the body. It is speculated that the occurrence of echinococcosis is closely related to a change in the intestinal microbiota, which is also a future research topic for echinococcosis pathogenesis and treatment.

The results revealed that there was no change in the diversity of gut microbiota in each group at the phylum level, but there were some changes in the populations of the gut microbiome at the genus and species levels among the AE, CE, and healthy groups. The differential bacteria in this study belonged to the following four phyla: Firmicutes, Proteobacteria, Bacteroidetes, and Actinobacteria. In the comparison among AE, CE, and healthy groups, *Rothia*, *Veillonella*, *Streptococcus*, and *Alistipes* species were detected in the feces of AE patients. These bacteria play a role in the pathogenesis of AE. For example, *Rothia* produces short-chain fatty acid (SCFAs), promoting mucus secretion—especially butyric acid, which affects colon movement and has anti-inflammatory properties (Wrzosek et al., 2013). *Veillonella* ferments pyruvate, lactic acid, malic acid, and oxalic acid and produces an endotoxin, which plays a pathogenic role in various mixed infections. Under normal circumstances, *S. salivarius* and *Streptococcus rheumaticus* colonize the oral cavity. These types of *Streptococcus* are harbored in the gut of AE patients, indicating that the oral bacterial community migrates during AE and their metabolites—such as butyric acid and propionic acid—increase along with the decomposed products of gut nutrients. *Alistipes* spp. produce succinic acid as the principal metabolic end-product of glucose fermentation (Parker et al., 2020; Radka et al., 2020). The compositional abundance of *Alistipes* in the feces seems to play a critical role in gut dysbiosis (Song et al., 2006). AE grows like a tumor, during which the abundance of *A. finegoldii* increases and has a protective role.

Carbohydrate Active Enzymes play a vital role in the lysis of complex carbohydrates such as cellulose, glycans, starches, and glycogen into components absorbed by the intestinal epithelium (Zhang et al., 2018). In this study, it was found that the gut microbiota had changed. Carbohydrate enzyme analysis revealed that the GH enzyme changes were obvious in the AE patients, and the GT change was obvious in CE patients. Moreover, the biosynthesis of glycoside compounds occupies an important position in the field of synthesis, affecting the metabolism of glycosides in the gut, which might affect the body’s immune mechanism. The biosynthesis of cell-surface glycochains is mainly catalyzed by the superfamily of glycosyltransferases. The expression of glycosyltransferase and abnormal glycochain structures regulate the interaction between tumor cells and the extracellular matrix, which is a common feature of the occurrence, development, and metastasis of malignant tumors (Oliveira-Ferrer et al., 2017). Glycoside hydrolase degrades natural polysaccharides and hydrolyzes various sugar-containing compounds. Glycosyltransferases exert their effect on stimulating various biological processes such as the growth of beneficial bacteria in the gut. Some can catalyze cellulose degradation (Sun et al., 2020). The role of Glycoside hydrolase and Glycosyltransferases in the pathogenesis of echinococcosis is a complex problem, which needs to be studied in the future.

Pathologically, the main disorders of the human gut microbiome are decreased bacterial diversity, a reduction in beneficial bacteria, and an increase in potentially pathogenic bacteria, which can lead to disease or might aggravate a disease process. Bao et al. (2018) found that *Eisenbergiella* and *Parabacteroides* were enriched at the genus level in mice infected with *E. granulosus*. Functional analysis indicated that seven pathways were altered in the *E. granulosus* infection group compared to levels in the uninfected group (Seki et al., 2007). In our study, the differences in the gut microbiome composition of AE, CE, and healthy individuals might be preliminary and requires further investigation. Our results revealed that the gut microbiomes in the AE, CE, and healthy groups were different. The differences in the roles of these bacteria in alveolar and CE will be a focus of our future research. Berrilli et al. (2012) suggested that the gut microbiota might not only drive the susceptibility to parasites but also the outcome of parasite infection. Moreover, differences in microbiota signatures could reflect the severity of parasite infections. It is certain that this field of research can enrich the diagnostic prospects of echinococcosis in the future. This study revealed the gut microbial changes in patients with echinococcosis using metagenomic next-generation sequencing and bioinformatics analysis. Changes in the intestinal microbiota of patients with echinococcosis might be related to the occurrence and development of echinococcosis. An analysis of the CAZymes database showed that carbohydrate enzymes can differ depending on the gut microbiome in patients with AE and CE. Blood biochemical indicators also differed in relation to the gut microbiome characteristics. Increased gut–liver axis permeability, as well as other bacterial metabolites (e.g., endotoxins, carbohydrate-active enzymes, etc.) and fecal bile acids are important targets affecting the progression of echinococcosis, and fecal microbial metabolites such as SCFAs should be the focus of future research.

Although there were only seven AE patients and six CE patients in this study, we found differences in gut microbiota in both AE and CE patients and some questions were raised. This represents a good beginning for future studies into the relationship between the gut microbiota and echinococcosis. In the next phase of this research, we will increase the sample size to study the gut microbiota of echinococcosis patients, with a view toward understanding the pathogenesis of AE and CE from the perspective of gut microbiota and identifying the bacterial markers associated with disease specificity.

## Data Availability Statement

The datasets presented in this study can be found in online repositories. The names of the repository/repositories and accession number(s) can be found below: National Microbiology Data Center of China, NMDC40021950.

## Ethics Statement

The studies involving human participants were reviewed and approved by P-SL-2018011. The patients/participants provided their written informed consent to participate in this study.

## Author Contributions

DC designed and wrote the manuscript. MP and DW collected the samples and conducted the sequencing experiment. GC and XP analyzed the experiment raw data. KX performed the statistical analysis. HF revised the manuscript. All authors revised the final manuscript and approved the submitted version.

## Conflict of Interest

The authors declare that the research was conducted in the absence of any commercial or financial relationships that could be construed as a potential conflict of interest.

## Publisher’s Note

All claims expressed in this article are solely those of the authors and do not necessarily represent those of their affiliated organizations, or those of the publisher, the editors and the reviewers. Any product that may be evaluated in this article, or claim that may be made by its manufacturer, is not guaranteed or endorsed by the publisher.

## Footnotes

1
http://bio-bwa.sourceforge.net/

2
www.mothur.org/

3
http://bcb.unl.edu/dbCAN2/

## References

[B1] Anter A. Mohamed Abd El-Ghany M. A. El-Dahab M. A. (2020). Does curcumin have a role in the interaction between gut microbiota and schistosoma mansoni inmice? *Pathogens* 9:676. 10.3390/pathogens9090767 32961786 PMC7558489

[B2] Bajaj J. S. Heuman D. M. Hylemon P. B. Sanyal A. J. White M. B. Monteith P. (2014). Altered profile of human gut microbiome is associated with cirrhosis and its complications. *J. Hepatol.* 60 940–947. 10.1016/j.jhep.2013.12.019 24374295 PMC3995845

[B3] Bao J. L. Zheng J. H. Wang Y. Z. Zheng X. T. He L. Qi W. J. (2018). Echinococcus granulosus infection results in an increase in *Eisenbergiella* and *Parabacteroides* genera in the gut of mice. *Front. Microbiol.* 9:2890. 10.3389/fmicb.2018.02890 30555437 PMC6281689

[B4] Berrilli F. Cave D. D. Cavallero S. D’Amelio S. (2012). Interactions between parasites and microbial communities in the human gut. *Front. Cell Infect. Microbiol.* 2:141. 10.3389/fcimb.2012.00141 23162802 PMC3499702

[B5] Bhat M. Arendt B. M. Bhat V. Renner E. L. Humar A. Johane P. (2016). Implication of the intestinal microbiome in complications of cirrhosis. *World J. Hepatol.* 8 1128–1136. 10.4254/wjh.v8.i27.1128 27721918 PMC5037326

[B6] Burcelin R. Courtney M. Amar J. (2015). Gut microbiota and metabolic diseases: from pathogensis to therapeutic perspective. *Metab. Gut Microb. Nutr. Dis.* 2015 199–234. 10.1007/978-1-4471-6539-2_11

[B7] Chinen T. Rudensky A. Y. (2012). The effects of commensal microbiota on immune cell subsets and inflammatory responses. *Immunol. Rev.* 245 45–55. 10.1111/j.1600-065X.2011.01083.x 22168413

[B8] Costea P. I. Zeller G. Sunagawa S. Pelletier E. Alberti A. Levenez F. (2017). Towards standards for human fecal sample processing in metagenomic studies. *Nat. Bioechnol.* 35 1069–1076. 10.1038/nbt.3960 28967887

[B9] Dai T. Li L. (2021). Gut microbiota and chronic liver disease. *Chin. J. Integrat. Tradit. Western Med. Liver Dis.* 31 769–773.

[B10] Dapito D. H. Mencin A. Youn-Gwak G. Y. Pradere J. P. Jang M. K. Mederacke I. (2012). Promotion of hepatocellular carcinoma by the intestinal microbiota and TLR4. *Cancer Cell.* 2012 504–516. 10.1016/j.ccr.2012.02.007 22516259 PMC3332000

[B11] Echinococcosis Surgical Committee Surgeons Branch Chinese Medical Association (2019). Expert consensus on diagnosis and treatment of hepatic cystic and alveolar echinococcosis. *Chin. J. Digest. Surgery* 18 711–721.

[B12] Eckburg P. B. Bik E. M. Bernstein C. N. Purdom E. Dethlefsen L. Sargent M. (2005). Diversity of the human intestinal microbial flora. *Science* 308 1635–1638. 10.1126/science.1110591 15831718 PMC1395357

[B13] Gui Q. Jin H. Zhu F. Lu H. Zhang Q. Xu J. (2021). Gut microbiota signatures in *Schistosoma japonicum* infection induced liver cirrhosis patients: a case-control study. *Infect. Dis. Poverty* 10:43. 10.1186/s40249-021-00821-8 33771232 PMC8004463

[B14] Kern P. Menezes da Silva A. Akhan O. Mullhaupt B. Vizcaychipi K. A. Budke C. (2017). The echinococcoses: diagnosis, clinical management and burden of disease. *Adv. Parasitol.* 96 259–369. 10.1016/bs.apar.2016.09.006 28212790

[B15] Martin R. Nauta A. J. Ben Amor K. Knippels L. M. J. Knol J. Garssen J. (2010). Early life: gut microbiota and immune development in infancy. *Beneficial Microbes* 1 367–382. 21831776 10.3920/BM2010.0027

[B16] McManus D. P. Gray D. J. Zhang W. Yang Y. (2012). Diagnosis, treatment, and management of echinococcosis. *BMJ* 344:e3866. 10.1136/bmj.e3866 22689886

[B17] Menzel P. Ng K. L. Krogh A. (2016). fast and sensitive taxonomic classification for metagenomocs with kaiju. *Nat. Commun.* 7:11257. 10.1038/ncomms11257 27071849 PMC4833860

[B18] Minemura M. Shimizu Y. (2015). Gut microbiota and liver diseases. *World J. Gastroenterol.* 21 1691–1702. 10.3748/wjg.v21.i6.1691 25684933 PMC4323444

[B19] Oliveira-Ferrer L. Legler K. Milde-Langosch K. (2017). Role of protein glycosylation in cancer metastasis. *Sem. Cancer Biol.* 44 141–152. 10.1016/j.semcancer.2017.03.002 28315783

[B20] Parker B. J. Wearsch P. A. Veloo A. C. M. Rodriguez-Palacios A. (2020). The genus alistipes: gut bacteria with emerging implications to inflammation, cancer, and mental health. *Front. Immunol.* 2020:906. 10.3389/fimmu.2020.00906 32582143 PMC7296073

[B21] Qin J. J. Li R. Q. Raes J. Manimozhiyan Arumugam M. Burgdorf K. S. Chaysavanh Manichanh C. (2010). A human gut microbial gene catalog established by metagenomic sequencing. *Nature* 464 59–65. 10.1038/nature08821 20203603 PMC3779803

[B22] Qin J. J. Li Y. R. Cai Z. M. Li S. H. Zhu J. F. Fan Zhang F. (2012). A metagenome-wide association study of gut microbiota in type 2 diabetes. *Nature* 490 55–60. 10.1038/nature11450 23023125

[B23] Qin N. Yang F. Li A. Prifti E. Chen Y. Shao L. (2014). Alterations of the human gut microbiome in liver cirrhosis. *Nature* 513 59–64. 10.1038/nature13568 25079328

[B24] Radka C. D. Frank M. W. Rock C. O. Yao J. W. (2020). Fatty acid activation and utilization by *Alistipes finegoldii*, a representative bacteroidetes resident of the human gut microbiome. *Mol. Microbiol.* 113:807. 10.1111/mmi.14445 PMC7176543 31876062

[B25] Ren W. X. Xiao X. S. Wen H. (2007). Advancement in imaging diagnosis and treatment of hepatic alveolar echinococcosis. *Chin. J. Interv. Imaging Ther.* 4 314–317.

[B26] Schachter J. de Oliveira D. A. da Silva C. M. de Barros Alencar A. C. M. Duarte M. da Silva M. M. P. (2020). Whipworm infection promotes bacterial invasion, intestinal microbiota imbalance, and cellular immunomodulation. *Infect. Immun.* 88 e642. 10.1128/IAI.00642-19 31843966 PMC7035941

[B27] Schneeberger P. H. Coulibaly J. T. Panic G. Daubenberger C. Gueuning M. Frey J. E. (2018). Investigations on the interplays between *Schistosoma mansoni*, praziquantel and the gut microbiome. *Parasit Vectors.* 11:168. 10.1186/s13071-018-2739-2 29530088 PMC5848565

[B28] Seki E. DeMinicis S. Osterreicher C. H. Kluwe J. Osawa Y. Brenner D. A. (2007). TLR4 enhances TGF-beta signaling and hepatic fibrosis. *Nat. Med.* 2007 1324–1332. 10.1038/nm1663 17952090

[B29] Song Y. L. Könönen E. Rautio M. Liu C. X. Bryk A. Eerola E. (2006). *Alistipes onderdonkii* sp. nov. and *Alistipes shahii* sp. nov., of human origin. *Int. J. Syst. Evolu. Microbiol.* 56 1985–1990. 10.1099/ijs.0.64318-0 16902041

[B30] Sun H. Yang G. Cao R. Mao X. Liu Q. (2020). Expression and characterization of a novel glycoside hydrolase family 46 chitosanase identified from marine mud metagenome. *Int. J. Biol. Macromol.* 2020 904–910. 10.1016/j.ijbiomac.2020.05.147 32446901

[B31] Unger M. Spiegel J. Dillmann K. U. (2016). Short chain fatty acids and gut microbiota differ between patients with Parkinson’s disease and age-matched controls[J]. *Parkins. Related Dis.* 32 66–72. 10.1016/j.parkreldis.2016.08.019 27591074

[B32] Vijay-Kumar M. Aitken J. D. Carvalho F. A. Cullender T. C. Mwangi S. Srinivasan S. (2010). Metabolic syndrome and altered gut microbiota in mice lacking toll-like receptor 5. *Science* 328 228–231. 10.1126/science.1179721 20203013 PMC4714868

[B33] Wang L. Liu Y. Zhu J. Zhong Y. Li S. Xu J. (2017). Role of short-chain fatty acids in disease treatment. *World J. Chin. Digest.* 25 1179–1186.

[B34] Wang Q. Huang Y. Huang L. Yu W. J. He W. Zhong B. (2014). Review of risk factors for human echinococcosis prevalence on the qinghai-tibet plateau, china: a prospective for control options. *Infect. Dis. Poverty* 3:3. 10.1186/2049-9957-3-3 24475907 PMC3910240

[B35] Wang X. K. Xu X. Q. Xia Y. (2017). Further analysis reveals new gut microbiome markers of type 2 diabetes mellitus. *Antonie Van Leeuwenhoek* 110 445–453. 10.1007/s10482-016-0805-3 27943013

[B36] Wen H. Vuitton L. Tuxun T. H. J. Li J. Vuitton D. A. Zhang W. B. (2019). Echinococcosis: advances in the 21st century. *Clin. Microbiol. Rev.* 32:e75. 10.1128/CMR.00075-18 PMC6431127 30760475

[B37] Wrzosek L. Miquel S. Noordine M. L. Bouet S. Joncquel Chevalier-Curt M. Robert V. (2013). *Bacteroides* thetaiotaomicron and *Faecalibacterium prausnitzii* influence the production of mucus glycans and the development of goblet cells in the colonic epithelium of a gnotobiotic model rodent. *BMC Biol.* 11:61. 10.1186/1741-7007-11-61 23692866 PMC3673873

[B38] Wu Y. H. Luo Z. Liu C. T. (2021). Variations in fecal microbial profiles of acute exacerbations and stable chronic obstructive pulmonary disease. *Life Sci.* 265:118738. 10.1016/j.lfs.2020.118738 33181175

[B39] Xu F. Cheng R. T. Miao S. H. Zhu Y. W. Sun Z. Qiu L. Y. (2020). Prior toxoplasma gondii infection ameliorates liver fibrosis induced by schistosoma japonicum through inhibiting th2 response and improving balance of intestinal flora in mice. *Int. J. Mol. Sci.* 21:2711. 10.3390/ijms21082711 32295161 PMC7216211

[B40] Yin Y. Mao X. Yang J. Chen X. Mao F. Xu Y. (2012). dbCAN: a web resource for automated carbohydrate-active enzyme annotation. *Nucleic Acids Res.* 40 W445–W451. 22645317 10.1093/nar/gks479 PMC3394287

[B41] Yooseph S. Kirkness E. F. Tran T. M. Harkins D. M. Jones M. B. Torralba M. B. (2015). Stool microbiota composition is associatedwith the prospective risk of Plasmodium falciparum infection. *BMC Genom.* 16:631. 10.1186/s12864-015-1819-3 26296559 PMC4546150

[B42] Zhang H. Yohe T. Huang L. Entwistle S. Wu P. Yang Z. (2018). dbCAN2: a meta server for automated carbohydrate-active enzyme annotation. *Nucleic Acids Res.* 46 W95–W101. 10.1093/nar/gky418 29771380 PMC6031026

[B43] Zhang X. Zhong H. Li Y. Shi Z. Ren H. Zhang Z. (2021). Sex- and age-related trajectories of the adult human gut microbiota shared across populations of different ethnicities. *Nat. Aging* 1:87-100. 10.1038/s43587-020-00014-2 37118004

[B44] Zhao Y. Yang S. Li B. Li W. Wang J. Chen Z. (2019). Alterations of the mice gut microbiome *via Schistosoma japonicum* ova-induced granuloma. *Front. Microbiol.* 10:352. 10.3389/fmicb.2019.00352 PMC6411663 30891012

[B45] Zhou L. X. Han T. Liu B. W. Gao Y. T. Han H. Y. (2017). A preliminary analysis of composition and structure of intestinal microbiota in patients with liver cirrhosis or hepatocellular carcinoma. *J. Clin. Hepatol.* 33 1740–1744. 10.3969/j.issn.1001-5256.2017.09.022

